# Color Range and Color Distribution of Healthy Human Gingiva: a Prospective Clinical Study

**DOI:** 10.1038/srep18498

**Published:** 2015-12-22

**Authors:** Daniel K. Ho, Razvan Ghinea, Luis J. Herrera, Nikola Angelov, Rade D. Paravina

**Affiliations:** 1 Department of Periodontics, School of Dentistry, University of Texas Health Science Center at Houston, Texas, USA; 2 Department of Optics, Faculty of Science, University of Granada, Granada, Spain; 3 Department of Computer, Architecture and Computer Technology, University of Granada, Granada, Spain; 4 Department of Restorative Dentistry and Prosthodontics, School of Dentistry, University of Texas Health Science Center at Houston, Texas, USA

## Abstract

The aim of this study is to compile a comprehensive database on color range and color distribution of healthy human gingiva by age, gender and ethnicity. Spectral reflection of keratinized gingiva at upper central incisors was measured by spectroradiometer and converted into CIELAB values. Lightness range (ΔL*) for all groups together was 26.8. Corresponding a* (green-red) and b* (blue-yellow) ranges (Δa* and Δb*) were 18.3 and 13.0. Significant differences (p < 0.05) were recorded by age for L* and a* coordinates, by gender for b* coordinate, and by ethnicity for L*, a* and b* coordinates. R^2^-values between color coordinates were 0.01 (L*/a*), 0.03 (L*/b*), and 0.12 (a*/b*). The smallest color differences were recorded between age groups 46–60 and 60 + (ΔE* = 0.9), and between Caucasians and Hispanics (ΔE* = 1.1). Color difference by gender was 1.3. When total L*a*b* ranges were divided into four equal segments, 51.7% of subjects had L* value within the third segment (from lightest to darkest), 47.1% had a* value within the third segment (from less red to redder), and 59.3% had b* value within the second segment (from less yellow to yellower). It was found that ethnicity and age had statistically significant influence on the color of human gingiva.

What color is the human gingiva? It is frequently described as “coral pink” and is dependent upon the thickness of epithelium, the degree of keratinization, the magnitude of pigmentation, and the underlying vascularization^1^. Accurate tooth color selection is of paramount importance in rendering esthetic dental therapy to patients. While advances in “white esthetics” for teeth are evident, the development of gingival “pink esthetics” lags behind. Understanding of pink esthetics is therefore critical because it will allow clinicians to better match the gingival portion of the dental prostheses to the soft tissues.

Typically, narrative approach is used when describing gingival color in studies, making it impossible to compare or combine outcomes of studies in a scientifically meaningful manner^2^. To date, only a few studies reported on color of gingival shade guides and gingival color matching. One study investigated the coverage errors of two commercial gingival shade guides in different ethnic groups and reported that both guides had high coverage errors^3^. Even when combining the two guides the smallest coverage error found was still much greater than the color acceptability threshold of ΔE = 4.6^4^, resulting in clinically unacceptable color match^3^,^5^. When several different gingival porcelain shade guides were examined, it was reported that they did not provide adequate color selection in matching pink porcelain to human gingiva^6^. With a limited research in this area, the literature suggests that the current gingival shade guides do not provide adequate and accurate color match to human gingiva.

A gingival shade guide ought to be developed based on the true representative colors of human gingiva. The color of human oral tissues was first described in 1950s^7^,^8^. Other methods have been developed to describe color of human gingiva, including visual assessment using Munsell system and photometric evaluation^9^,^10^,^11^,^12^,^13^,^14^,^15^,^16^. However, a systematic review reported some drawbacks in these studies^2^.

Several more recent studies have utilized The Commission Internationale de l′Eclairage, L, a*, b* (CIELAB) color notation system for assessment of color of human gingiva^17^,^18^. CIELAB is a 3-dimensional system with L* representing lightness, coordinate a* representing a measure of greenness (negative a*) or redness (positive a*), and coordinate b* representing a measure of blueness (negative b*) or yellowness (positive b*)^19^. CIELAB system allows the calculation of Euclidean distance (ΔE*), which is a measure of color difference between 2 points in the 3-dimensional color space that helps clinicians to evaluate degree of color matching between the shade tab and the color of the object (e.g. gingiva)^20^. One study investigated the color distribution of human gingiva in a cohort of 362 Taiwanese by analyzing CIELAB values obtained from spectrophotometry^17^. Based on their analysis, the study proposed a gingival shade guide containing 10 shade tabs; however, the results of this study may only be applied to Asian ethnicity^17^. Others have recently conducted a pilot study to evaluate the effect of race, age and gender on the CIELAB values of gingival colors in 120 subjects using spectroradiometry^18^. The group reported that L*a*b* values were significantly affected by gender and race but not age, and with clustering analysis they identified three clusters in these subjects for three gingival tones^18^. However, the small sample size might be seen as a limitation of this study^18^.

There is no doubt that CIELAB color coordinates are beneficial for characterizing color ranges and distribution of human gingiva. However, if one would need to generate data that can be used to formulate gingival color using corresponding dental materials, it would be necessary to collect data on reflection values throughout the visible spectra range (reflection curves), as they are fundamental optical property and the actual fingerprint of a color. The aim of this study was to compile a comprehensive database on fundamental optical properties, color range and color distribution of human gingiva by age, gender and ethnicity, and analyze the data collected in a large cohort of subjects from different age, gender and ethnic groups. The null hypothesis was that there were no differences in gingival color based on age, gender and ethnicity.

## Results

Age, gender and ethnic distribution of the subjects are presented in Table 1. There were 41% males and 59% females, with ethnic breakdown of 18% African-Americans, 23% Asians, 34% Caucasians, and 25% Hispanics.

**Figure 1:**
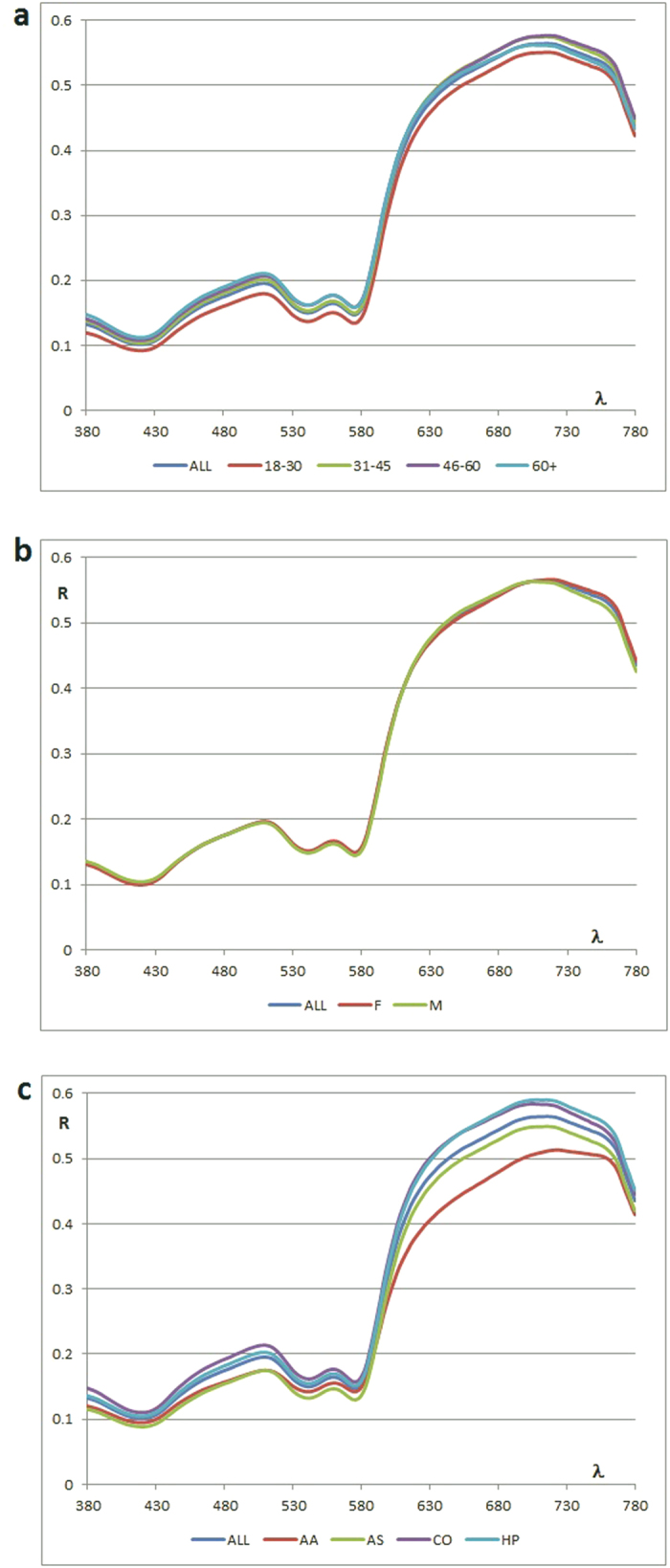
Reflection curves: (a) by age; (b) by gender; and (c) by ethnicity.

**Figure 2:**
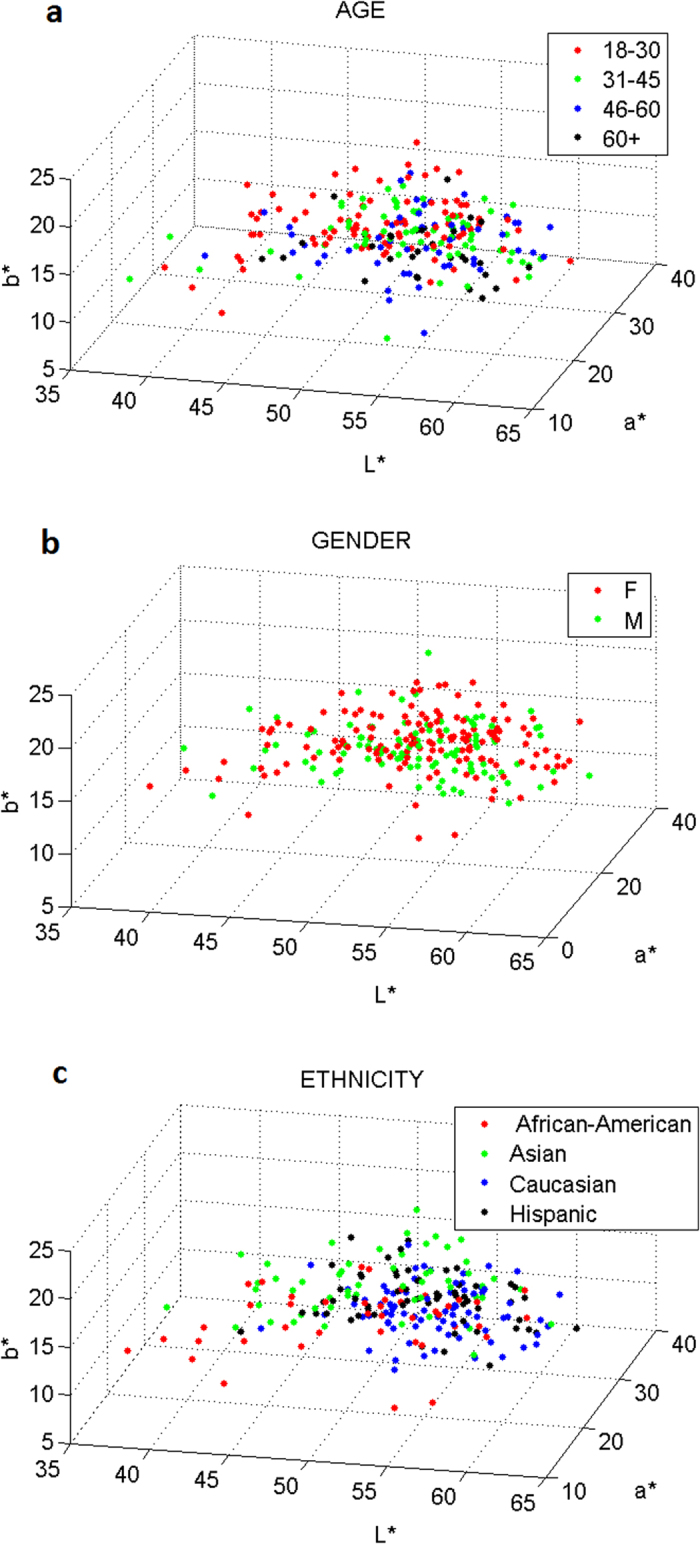
L*a*b* distribution: (a) by age; (b) by gender; and (c) by ethnicity.

Reflection curves by age, gender and ethnicity are shown in Fig. 1. Means and ranges of L*, a* and b* values for each subject group were reported in Table 2. Lightness range, ΔL*, for all subjects was 26.8. Corresponding a* and b* ranges (Δa* and Δb*) were 18.3 and 13.0, respectively. The distribution of color coordinates is presented in Fig. 2 and Table 3. Significant differences (p < 0.05) were recorded by age for L* and a* coordinates, by gender for b* coordinate, and by ethnicity for L*, a* and b* coordinates. R^2^ values between color coordinates were 0.01 (L*/a*), 0.03 (L*/b*), and 0.12 (a*/b*).

The greatest gingival color variations amongst the four age groups was between Group 1 (age 18-30) and Group 4 (age 60+) with ΔE* of 3.9, followed by that of Group 1 and Group 3 (age 46–60) with ΔE* of 3.5 (Table 4). The ΔE* for gingival color variation between females and males was 1.3. Evaluation of gingival color in different ethnic groups showed that ΔE* value was highest when comparing African-Americans to Caucasians (ΔE* = 5.0), followed by African-Americans versus Hispanics (ΔE* = 4.9), African-Americans versus Asians (ΔE* = 4.7), Asians versus Caucasians (ΔE* = 4.3), Asians versus Hispanics (ΔE* = 1.4), and Caucasians versus Hispanics (ΔE* = 1.1).

## Discussion

The null hypothesis that there were no differences in gingival color basing on age, gender and ethnicity has been rejected as significant differences (p < 0.05) in one or more color coordinates were recorded by age, gender, and ethnicity. Lightness (ΔL*) ranged the most, followed by the Δa* and Δb* coordinate ranges, respectively. However, R^2^-values between the color coordinate pairs were very low.

The study that was used for threshold comparisons in this paper reported CIELAB 50:50% acceptability threshold of ΔE* = 4.6 basing on color differences of denture-based acrylic resins upon aging^4^. Another study evaluated color differences of a series of human gingival photographs altered in Photoshop and reported acceptability threshold of ΔE* = 3.1 basing on acceptance of all observers^21^. Based on the 50:50% acceptability threshold of ΔE* = 4.6 for gingiva^4^, all age-dependent differences in the color of human gingiva reported in this study were below the 50:50% acceptability threshold of ΔE* = 4.6. The gingiva becomes lighter with age – the L* values were higher in older subjects when all ethnic groups were considered together. As far as color differences between different ethnic groups were concerned, the values above the ΔE* = 4.6 threshold were recorded between African-Americans and all other ethnic groups, while color differences close to this threshold were found between Asians and Caucasians. Lastly, gingival color did not vary by gender. Reflection curves presented in Fig. 1 perfectly illustrated the origin and magnitude of color differences. It is very interesting that the overall shape and peaks (per wavelengths, nanometers) of all reflection curves were almost identical.

Present findings on ethnicity as a factor contributing to differences in L*a*b* is in agreement with published data^18^. The differences in gingival color in different ethnicities are likely due to the degree of oral melanotic pigmentations across different ethnic groups^7^,^8^,^22^. A handful of studies have also examined the effect of gender and age on color of human gingiva; however, no consistent results have been obtained^9^,^11^,^17^,^18^. In this study, there were no significant differences in gingival color between men and women, but color varied by age and ethnicity. Graphic representation of color distribution in CIELAB system (Fig. 2) also provides a good tool for understanding the differences by age, gender and ethnicity.

One study reported that gender but not age had significant impact on the L*, a* and b* values of human gingiva^18^. In the same study, six subjects (three males and three females) were recruited for each of the four ethnic groups (Caucasians, African-Americans, Asian/Pacific Islander, and others) in each of the five age groups (18–29 years, 30–39 years, 40–49 years, 50–59 years, and 60–85 years), with a total of 120 subjects^18^. In the present study, we have recruited considerably greater number of subjects totaling to about twice the total number of subjects enrolled in the other study. Another study also showed that L*a*b* gingival color coordinates are significantly by gender but not age^17^. However, the subjects recruited in that study were limited to Taiwan. Major differences compared to this study were smaller sample size and narrower ethnic diversity of the subjects. Discrepancies in the reported outcomes on CIELAB values in different studies may be due to the use of different methodologies of color measurements, different sample size, and/or different sample population. Furthermore, data analyzed in previous studies^17^,^18^ were interpreted according to the tooth color acceptability for ΔE* of 5.5^5^ rather than the gingival color acceptability threshold of ΔE* = 4.6^4^.

In the present study, we have determined L*a*b* values from all 238 subjects and divided each of the L*a*b* ranges into four equal segments (Table 2). We have identified one segment for each of L*, a* and b* ranges that contained the highest percentage of subjects. This information might be very useful in the development gingival color shade guides based on the distribution of human gingiva.

Subjects in this study were students, staff, faculties and patients of The University of Texas at Houston School of Dentistry. Although difficult to accomplish, a more ideal population sample would consist of participants from a larger region with balanced ethnic diversity. Since the measured area of the gingiva used in our study was confined only to the keratinized gingiva on the buccal aspect of maxillary central incisor (same for other studies^17^,^18^), the obtained data may not be applicable to gingiva located in other areas of mouth. For example, individuals with very thin gingival biotype at mandibular anterior area may present with lighter gingival color because of the whiteness of the roots of mandibular anterior teeth showing through the thin overlying gingiva. Moreover, we noticed that certain individuals had distinct areas of pigmentation that was sharply defined by the surrounding lighter gingiva. As such, two distinct gingival colors (e.g. pigmented versus non-pigmented areas) may lay within the same field of our measurement, thereby leading us to over- or under-estimate the L*a*b* values of the gingiva. Lastly, the Asian group in our study included not just the Asian Pacific Islanders but also individuals from other parts of Asia such as India or Middle East who may present with different pigmented gingiva relative to Pacific Islanders. In spite of these limitations, our data on color range and color distribution of human gingiva can be used to explore the accuracy of existing gingival shade guides by comparing these guides to our clinical data. More importantly, our clinical data might aid in the development of more accurate gingival shade guides for clinical use.

Within the limitations of this study, it was concluded that ethnicity and age had statistically significant influence on color of human gingiva. Lightness increased, while a* and b* coordinate values decreased with age. Gingiva of African-American subjects was darker, less red and less yellow compared to other ethnicities. Significant color differences were recorded between African-American subjects and subjects of other ethnicities, followed by the difference between subjects of Asian and Caucasian ethnicity. No significant gender-dependent color differences were recorded.

## Methods

The study protocol was approved by Institutional Review Board (IRB) at The University of Texas Health Science Center at Houston (HSC-DB-13-0646). This study was carried out in accordance to the approved guidelines outlined by the IRB (HSC-DB-13-0646). A total of 238 subjects including faculties, staff, students and patients age 18 or older were recruited at The University of Texas at Houston School of Dentistry. Informed consents were obtained from all subjects. Each subject was informed of the purpose and the benefits of the study, as well as the procedure, time commitment, and discomforts related to the study. De-identified subjects’ information was kept confidential and they were given an option to withdraw from the study at any time.

Each subject filled a questionnaire containing information on demographics, medical and dental histories, and oral/social habits. Subjects were grouped according to age, gender, and ethnicities as follows: Group 1 (ages 18–30), Group 2 (ages 31–45), Group 3 (ages 46–60), Group 4 (ages 61+), Group M (male), Group F (female), Group AA (African-American), Group AS (Asian), Group CA (Caucasian), and Group HP (Hispanic). Subjects were screened for gingival health at either upper right or left central incisors according to the inclusion and exclusion criteria outlined in Table 5. The gingival color of the subjects was measured using a spectroradiometer PR-670 together with MS-75 accessory lens (Photo Research, Chatsworth, CA) with measurement area of 6 mm in diameter. Each subject’s forehead and lower jaw were positioned on the optometry head frame attached to the optical table. Spectral reflections of keratinized gingiva 2-3 mm apical to mid-facial gingival margin of #8 or #9 were collected and the anonymized data were converted into CIELAB (Commission Internationale de L’Eclairage, L, a*, b*) values. Means and standard deviations were calculated. Color differences (ΔE*) among the mean values for each group were determined using the following equation^20^: ΔE* = [(ΔL*)^2^ + (Δa*)^2^ + (Δb*)^2^]^½^, where L*, a*, and b* corresponded to differences in lightness, green-red coordinate and blue-yellow coordinate, respectively.

Means and standard deviations were determined. Kruskal-Wallis test was used to compare L*a*b* values at α = 0.05. Clinical relevance of recorded color differences was additionally interpreted using the published value for acceptability threshold of ΔE* = 4.6^4^.

## Additional Information

**How to cite this article**: Ho, D. K. *et al.* Color Range and Color Distribution of Healthy Human Gingiva: a Prospective Clinical Study. *Sci. Rep.* **5**, 18498; doi: 10.1038/srep18498 (2015).

## Figures and Tables

**Table 1:** Subject distribution by age, gender and ethnicity: AA = African-American, AS = Asian, CA = Caucasian, HP = Hispanic.

Age	Male (n = 97)				Female (n = 141)			
	18–30	31–45	46–60	60+	18–30	31–45	46–60	60+
AA (n = 42)	3	2	3	5	9	7	13	0
AS (n = 54)	10	5	5	1	16	10	6	1
CA (n = 82)	15	9	2	9	14	11	14	8
HP (n = 60)	11	9	5	3	12	12	4	4

**Table 2:** L*a*b* ranges divided into 4 equal segments and percentage of subjects belonging to each segment (the highest percentages of subjects are underlined in corresponding L*, a* and b* segments).

L* Range/Percentage	a* Range/Percentage	b* Range/Percentage
37.2 to 43.8/5.9	13.4 to 17.9/5.5	9.2 to 12.3/8.8
43.9 to 50.5/23.1	18.0 to 22. 5/36.1	12.4 to 15.6/59.3
50.6 to 57.2/51.7	22.6 to 27.0/47.1	15.7 to 18.9/29.4
57.3 to 64.0/19.3	27.1 to 31.7/11.3	19.0 to 22.2/2.5

**Table 3:** Color coordinate values by group.

Group	Color coordinates					
	L*		a*		b*	
	Range	Mean (SD)	Range	Mean (SD)	Range	Mean (SD)
1	39.6–63.2	51.2 (5.2)	13.4–31.7	24.2 (3.4)	10.5–22.2	15.2 (2.0)
2	37.2–62.2	53.4 (5.2)	13.6–29.9	23.5 (3.2)	9.2–18.9	14.8 (1.8)
3	41.6–64.0	54.3 (4.8)	13.8–31.6	22.3 (3.6)	9.3–19.7	14.8 (2.1)
4	44.2–62.6	54.4 (4.2)	17.9–29.0	22.1 (2.7)	11.0–18.6	14.1 (2.0)
F	37.2–64.0	53.0 (5.3)	13.4–29.9	22.9 (3.4)	9.2–20.0	15.2 (2.0)
M	38.5–63.2	52.8 (5.0)	13.8–31.7	23.9 (3.2)	10.7–22.2	14.4 (1.9)
AA	37.2–61.7	50.6 (6.2)	13.4–27.2	20.4 (3.3)	9.2–17.5	14.3 (2.0)
AS	38.5–62.8	50.8 (5.5)	17.9–30.0	24.8 (3.1)	10.7–22.2	15.7 (2.1)
CA	43.2–64.0	54.7 (3.9)	13.8–29.0	23.3 (2.9)	11.0–19.7	14.4 (1.8)
HP	43.8–63.2	53.8 (4.4)	18.4–31.7	24.1 (3.1)	11.3–19.2	15.1 (1.7)
All	**37.2–64.0**	**52.9 (5.2)**	**13.4–31.7**	**23.3 (3.4)**	**9.2–22.2**	**14.9 (2.0)**

1 = age 18–30, 2 = age 31–45, 3 = age 46–60, 4 = age 60 + , M = males; F = females, AA = African-American, AS = Asian, CA = Caucasian, HP = Hispanic.

**Table 4:** Color differences (ΔE*) among groups.

Group	ΔE*
1 vs 2	2.2
1 vs 3	3.5
1 vs 4	3.9
2 vs 3	1.6
2 vs 4	2.1
3 vs 4	0.9
F vs M	1.3
AA vs AS	4.7
AA vs CA	5.0
AA vs HP	4.9
AS vs CA	4.3
AS vs HP	1.4
CA vs HP	1.1

1 = age 18–30, 2 = age 31–45, 3 = age 46–60, 4 = age 60 + , M = males; F = females, AA = African-American, AS = Asian, CA = Caucasian, HP = Hispanic.

**Table 5:**
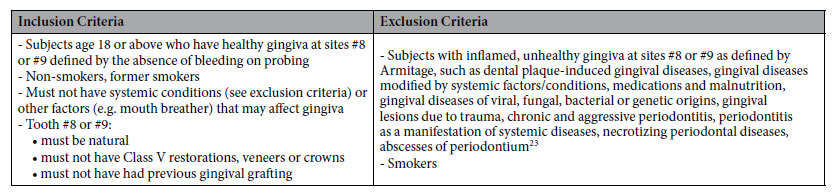
Inclusion and exclusion criteria.
